# The impact of older adults' volunteer motivation on successful aging: a moderated mediation model

**DOI:** 10.3389/fpubh.2026.1710061

**Published:** 2026-02-09

**Authors:** Jiao Mi, Rong Yuan, Yuwei Tan, Xue Li

**Affiliations:** 1 Neurological Intensive Care Unit, Deyang People's Hospital, Deyang, China; 2 Hepatological Surgery Department, Deyang People's Hospital, Deyang, China

**Keywords:** older adults, volunteer motivation, altruistic behavior, loneliness, successful aging inventory, moderating effects

## Abstract

**Objective:**

This study explores the relationship between volunteer motivation and successful aging among older adults, with a specific focus on the intermediary function of altruistic behavior. It also examines whether loneliness moderates the altruism-mediated pathway within this association.

**Methods:**

A cross-sectional study was carried out between January and May 2024, involving older adults from five regions in Sichuan Province, China. Participants completed a battery of instruments, including a general information form, the Volunteer Function Inventory (VFI), the Successful Aging Inventory (SAI), the Self-Report Altruism Scale–Distinguished by the Recipient (SRAS-DR), and the Chinese adaptation of the De Jong Gierveld Loneliness Scale (DJGLS). Data analysis was performed using SPSS along with the PROCESS macro to examine the hypothesized linkages and mediation/moderation effects.

**Results:**

Older adults' volunteer motivation significantly and positively predicted both successful aging and altruistic behavior. Altruistic behavior served as a partial mediator in the link between volunteer motivation and successful aging. Moreover, loneliness moderated the latter stage of the mediating pathway, attenuating the positive predictive effect of altruistic behavior on successful aging.

**Conclusion:**

With China's aging population continuing to grow, society and families should actively address this challenge by rejecting the traditional notion of the “uselessness of old age,” cultivating and supporting seniors' motivation to engage in voluntary work, and recognizing their ongoing value and contributions to community well-being. Greater consideration should also be given to psychological well-being, particularly alleviating feelings of loneliness. By designing appropriate volunteer programs that match their capabilities, we can strengthen their sense of social engagement and further facilitate successful aging.

## 1 Introduction

The rapid advancements witnessed in the global economy, coupled with the substantial improvements in medical care, have made global population aging increasingly prominent, and it is exhibiting an irreversible trend (1). China has a large older adult population, and the rate of aging is increasing. The total population was made up, by the end of 2023, of 21.1% individuals aged 60 years and older. Projections indicate that by 2035, the older adult population will reach 400 million—over 30% of the total—signifying the country's transition into a stage of profound aging (2). Consequently, addressing population aging has become a vital priority within China's overall development agenda. Despite this urgency, numerous challenges persist, including an underdeveloped health management system, sluggish growth of the aging-related industry, and pronounced disparities in older adult care services between urban and rural areas. For older adults themselves, achieving successful aging can foster greater enthusiasm for social engagement, strengthen health awareness, and enhance subjective well-being. From a societal perspective, enabling older adults to continue contributing to public affairs by drawing on their personal expertise can transform the risks of aging into a “longevity dividend.” Furthermore, through improved self-care and self-regulation, older adults can maintain good physical and mental health, thereby alleviating the medical and caregiving burdens on both society and families (3). In this context, addressing the challenges of population aging hinges on a deeper exploration of the theoretical and practical implications of successful aging.

The notion of successful aging was initially proposed by Havighurst in 1961. He proposed that “longevity” and “life satisfaction” are the primary criteria for evaluating successful aging among older adults (4). With further research, Rowe and Kahn (5) advanced a widely recognized three-dimensional model, defining successful aging through physiological, psychological, and social dimensions. Ryff (6) identified six positive predictors of successful aging in older adults: self-acceptance, positive relationships, autonomy, environmental mastery, purpose in life and personal growth. Further extending the theoretical framework, Professor Flood developed a middle-range theory of care specifically tailored to the aging process and, based on this theory, designed the Successful Aging Self-Assessment Scale. This instrument evaluates successful aging across five dimensions: functional coping, internal factors and meaning in life, transcendence of aging, sense of legacy, and spirituality (7). The scale consists of 20 items and demonstrates good internal consistency, with a Cronbach's α coefficient of 0.86. In China, the version most commonly used in research is the Chinese adaptation translated by Cheng (8), which has been validated as an accurate and comprehensive measure for assessing successful aging in the domestic context. Successful aging refers to a multidimensional construct that encompasses sufficient social support, the absence of limitations in activities of daily living or instrumental activities of daily living, no diagnosed mental illness in the past year, the lack of severe cognitive impairment or pain interfering with normal functioning, a high sense of well-being, sound physical and mental health, and self-perceived successful aging (9).

Social exchange theory posits that older adults benefit from active participation in social activities such as volunteer work and community engagement (10). These altruistic behaviors not only help maintain physical functioning but also foster a positive mindset, enhance self-worth, and support the achievement of successful aging. Accordingly, altruistic behavior can serve as a mediating variable between volunteer motivation and successful aging. An intervention study conducted in Germany with 280 community-dwelling older adults revealed that those who received encouragement for volunteer participation demonstrated higher levels of volunteer engagement and were more likely to achieve successful aging (11). Similarly, a cross-sectional study in China employing a multi-stage stratified sampling method surveyed 318 adults aged 60 and above in Jinzhou, showing that volunteer service was a positive predictor of successful aging and was positively correlated with health, well-being, and longevity (12). Therefore, examining the pathways through which volunteer motivation influences successful aging holds important implications for promoting older adults' well-being. Furthermore, the prevalence of feelings of loneliness in later life represents a significant public health concern. A survey of 428 older adults residing in nursing homes across different regions of China indicated a negative correlation between loneliness and successful aging. The analysis suggested that negative psychological states such as loneliness can exacerbate sleep problems, impair cognitive function, and, over time, accelerate physical decline—creating a vicious cycle that undermines successful aging (13). Accordingly, an article proposing strategies for sustainable volunteer development emphasized the need to improve institutional support for older adult volunteering and to further investigate its underlying mechanisms to enhance successful aging (14). However, existing research has not yet explored the potential mediating effect of altruistic behavior or the moderating influence of loneliness within the linkage between older adults' volunteer motivation and successful aging. The theory of productive aging emphasizes the significance of involving older individuals in meaningful and productive roles, which allows them to make valuable social contributions and achieve personal fulfillment, thus promoting successful aging. Grounded in social exchange theory and productive aging theory, this study primarily examines the predictive effect of older adults' volunteer motivation on successful aging, the mediating role of altruistic behavior in the relationship between volunteer motivation and successful aging, and the moderating effect of loneliness on this mediating pathway. By exploring these mechanisms, this research aims to provide new insights for enhancing the well-being of older adults, such as promoting government and societal efforts to improve institutional systems for volunteer services, and creating sustainable and supportive social environments that foster successful aging through volunteer engagement.

### 1.1 Volunteer motivation and successful aging

The concept of volunteer motivation pertains to the internal psychological processes that initiate and sustain an individual's participation in volunteer activities, serving as a critical foundation for volunteer engagement (15). Numerous studies support a dichotomous framework for categorizing volunteer motivation, distinguishing between self-oriented and other-oriented (altruistic) motives, or between intrinsic and extrinsic motives (16, 17). Drawing on functional analysis theory, Clary et al. (18) identified six dimensions of volunteer motivation: learning and understanding, career development, value expression, self-enhancement, self-protection, and social interaction. Learning and understanding refers to volunteers' desire to acquire new knowledge or demonstrate skills not frequently used in daily life through their service. Career development reflects the expectation of gaining career-related experience. Value expression involves the wish to convey important personal values. Self-enhancement refers to the pursuit of psychological growth and personal development. Self-protection encompasses the aim of alleviating negative emotions or addressing personal problems. Finally, social interaction relates to the intention of strengthening one's social relationships through volunteer activities (19).

According to Maslow's hierarchy of needs, successful aging encompasses not only physical health and longevity but also psychological fulfillment and the continuation of social roles. Volunteer service offers older adults a platform to meet multiple needs. When these needs are fulfilled, they are more likely to perceive meaning and value in later life. Thus, a strong motivation for volunteer service can have a mutually reinforcing relationship with successful aging.

Empirical evidence further supports the influence of volunteer motivation on successful aging. A study of 498 middle-aged and older adults examined factors influencing volunteer participation and its impact on successful aging (20). The findings revealed that stronger volunteer motivation was associated with higher expectations and greater satisfaction with volunteer experiences. In turn, greater satisfaction predicted a stronger willingness to continue volunteering, which further enhanced successful aging. Therefore, positive volunteer motivation functions as a key protective factor for successful aging. A longitudinal study in Canada also found that volunteering is linked to successful aging in older adults (9). Collectively, these studies underscore that positive volunteer motivation is an important predictor of successful aging, thereby forming the basis for the first hypothesis.

Hypothesis 1: a significant positive correlation is present between older adults' volunteer motivation and successful aging.

### 1.2 The mediating role of altruistic behavior

Altruistic behavior refers to actions undertaken with the intention of benefiting others without any expectation of personal gain, representing a high level of moral development (21). Successful aging is shaped not only by volunteer motivation but also by altruistic behavior. According to self-actualization theory, for older adults, altruistic behavior can serve as a pathway to realizing self-worth, enabling them to pass on their experience, wisdom, and skills to others while contributing to society (22).

Volunteer motivation and altruistic behavior are also closely interrelated. In China's aging society, the disadvantaged status of older adults largely stems from societal prejudice toward this population, limited opportunities for social participation, and the deeply rooted traditional concept of family-based older adult care. These societal perceptions restrict opportunities for both social engagement and self-directed participation. As proposed by empowerment theory (23), the social environment plays a crucial role in supporting and empowering individuals. Through volunteer service, older adults can share their life experience, wisdom, and skills, thereby contributing to society and, at the same time, gaining opportunities to reassess their self-worth and capabilities. A study using questionnaires and semi-structured interviews to explore factors influencing older adults' volunteer motivation found that a high level of altruistic fulfillment significantly enhances their volunteer motivation (24). Another study investigating determinants of volunteer motivation among 770 volunteers also highlighted altruistic values as a key factor influencing older adults' volunteer motivation (25). Thus, positive volunteer motivation and altruistic behavior mutually reinforce each other.

Hypothesis 2: altruistic behavior mediates the relationship between older adults' volunteer motivation and successful aging.

### 1.3 The moderating effect of loneliness

Loneliness is an unpleasant and distressing psychological experience that can negatively impact successful aging in older adults. Although some older adults display similar levels of altruistic behavior, their levels of successful aging vary, and this variation may be shaped by a range of psychological and physiological factors. Loneliness, which often persists and intensifies with age, may be an important factor contributing to these differences (26). Loneliness can impair older adults' capacity to regulate their thoughts, emotions, and behaviors, which in turn reduces their participation in social activities and undermines successful aging. In summary, high levels of loneliness may weaken the positive influence of altruistic behavior on successful aging, whereas low levels of loneliness may strengthen this positive relationship. Thus, loneliness may serve as a negative moderator in the relationship between altruistic behavior and successful aging, forming the basis for Hypothesis 3.

Hypothesis 3: loneliness negatively moderates between altruistic behavior and successful aging.

### 1.4 The current study

**Figure 1:**
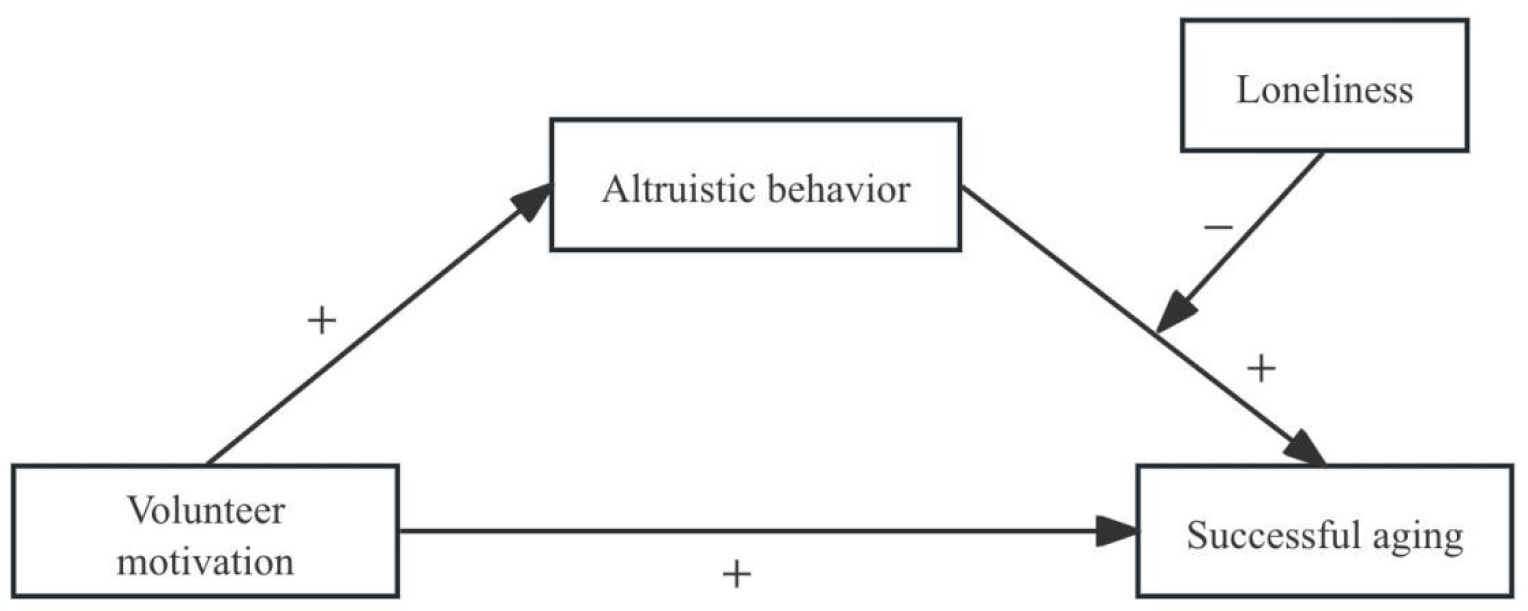
The proposed mediated moderation model.

Current literature lacks a comprehensive examination of the interplay between older adults' volunteer motivation, altruistic behavior, successful aging, and loneliness. Grounded in social exchange theory and productive aging theory, this study establishes a conceptual framework to investigate the complex mediating and moderating mechanisms linking volunteer motivation and successful aging in older individuals (see Figure 1). The specific aims include: (1) examining whether volunteer motivation significantly predicts successful aging; (2) assessing the mediating role of altruistic behavior in the relationship between volunteer motivation and successful aging; and (3) evaluating the moderating effect of loneliness on the association between altruistic behavior and successful aging.

## 2 Methods

### 2.1 Participants

The study was conducted from January to May 2024. Prior to data collection, the research team contacted and obtained permission from administrators of various organizations—including urban community committees, senior activity centers, older adult care institutions, and rural village committees—across several cities in Sichuan Province such as Deyang, Chengdu, and Nanchong. With their assistance, older adults who were interested in participating were identified and organized. A convenience sampling method was then used to enroll eligible individuals who met the study criteria. Eligibility requirements included being aged 60 years or older, possessing adequate visual, hearing, and verbal communication capabilities, as well as providing informed consent voluntarily. Exclusion criteria comprised a diagnosis of any psychiatric disorder or the presence of severe chronic conditions—including cardiovascular, cerebrovascular, or kidney diseases, or cancers—that could substantially impede participation.

A total of 330 questionnaires were distributed, and 313 valid responses were obtained after excluding invalid submissions, resulting in a valid response rate of 94.85%. The demographic characteristics of the participants were as follows: 172 males (55.0%) and 141 females (45.0%). Age distribution was: 71 participants (22.7%) aged 60–70 years, 175 (55.9%) aged 70–80 years, and 67 (21.4%) aged 80–90 years. Educational attainment included 75 participants (24.0%) with elementary education or below, 149 (47.6%) with junior or senior high school education, and 89 (28.4%) with education above the senior high school level. Regarding marital status, 218 participants (69.6%) were married, 51 (16.3%) widowed, and 44 (14.1%) divorced. In terms of residence, 195 participants (62.3%) lived in rural areas, and 118 (37.7%) lived in urban areas. Occupational backgrounds were as follows: 64 participants (20.5%) were farmers, 135 (43.1%) were self-employed, and 114 (36.4%) were former employees of public institutions.

### 2.2 Procedure

This study received ethical approval from the Ethics Committee of Deyang People's Hospital. Prior to data collection, all four researchers involved underwent standardized training. Participants were informed about the study's purpose, procedures, and questionnaire completion guidelines, and their written informed consent was obtained. Uniform instructions were provided throughout the process. For participants who experienced difficulty in completing the forms independently, the researchers assisted by asking each question individually and recording responses on their behalf. All questionnaires were collected and reviewed on-site for completeness; any missing responses were promptly supplemented. The study ensures strict confidentiality of all questionnaire data.

## 3 Measures

### 3.1 Volunteer service motivation scale

The Volunteer Service Motivation Scale, initially created by Clary and Snyder (27), was used to evaluate motivations behind volunteering. The Chinese adaptation of the scale was translated and validated for Chinese populations by Jiang in 2018 (19). This instrument consists of 18 items grouped into six dimensions—learning and understanding, career development, value expression, self-enhancement, self-protection, and social interaction—each containing three items. Responses are collected on a 5-point Likert scale from 1 (“strongly disagree”) to 5 (“strongly agree”), where higher scores reflect greater volunteer motivation. In this study, the total Cronbach's α for the scale was 0.916, indicating excellent internal consistency.

### 3.2 Self-report altruism scale distinguished by the recipient (SRAS-DR)

The SRAS-DR was initially introduced by Oda et al. (28) and later adapted into Chinese through translation and cultural validation by Guo et al. in 2017 (29). This scale includes 21 items categorized into three domains: kin altruism, peer altruism, and stranger altruism, each containing seven items. Participants respond using a 5-point Likert scale from 1 (“never”) to 5 (“very often”), where higher values indicate more frequent altruistic behaviors. In this study, the instrument exhibited high internal consistency, with a Cronbach's α of 0.931.

### 3.3 Loneliness scale

The Loneliness Scale was originally designed by De Jong-Gierveld et al. (30) to evaluate loneliness in older adults. Its Chinese version was translated and culturally adapted by Yang et al. in 2019 (31), with validation for local use. The scale includes 11 items measuring two dimensions: social loneliness (7 items) and emotional loneliness (4 items). Items are rated using a 3-point Likert scale from 1 (“No”) to 3 (“Yes”). Reverse scoring is applied to items 1, 4, 7, 8, and 11. Total scores range from 11 to 33, with higher values representing more severe loneliness. In this study, the scale showed good internal consistency, achieving a Cronbach's α of 0.883.

### 3.4 Successful aging scale

The Successful Aging Scale was originally constructed by Troutman et al. (32) based on Flood's middle-range theory of nursing (7). Its Chinese adaptation was rendered and validated by Cheng in 2014 (8). The scale contains 20 items organized into five domains: inner factors and meaning in life, functional coping mechanisms, transcendence of aging, sense of heritage, and spirituality. Responses are recorded on a 5-point Likert scale, with elevated scores reflecting a higher level of successful aging. In this study, the scale exhibited strong reliability, with a Cronbach's α of 0.898.

### 3.5 Data analysis

All statistical analyses were conducted with SPSS 26.0. Normality of the data distribution was evaluated using histograms and P–P plots. Continuous variables were summarized as mean ± standard deviation, and categorical variables were expressed as frequencies and percentages. Pearson correlation analysis was employed to assess the bivariate relationships among volunteer motivation, altruistic behavior, successful aging, and loneliness. Mediation and moderated mediation analyses were carried out using the PROCESS macro (version 4.1) developed by Hayes. Model 4 was utilized to examine the mediating role of altruistic behavior in the linkage between volunteer motivation and successful aging. Model 14 was used to test the moderating effect of loneliness on the latter part of the mediation model (33). Since significant differences in successful aging scores were detected across gender, age, and educational level (*F* = 0.197, *p* < 0.05; *F* = 11.454, *p* < 0.01; *F* = 4.132, *p* < 0.05), these variables were incorporated as covariates in the model. The bias-corrected percentile bootstrap approach with 5,000 resamples was used to estimate 95% confidence intervals. Effects were considered statistically significant when the confidence intervals did not include zero (34). A significance threshold of α = 0.05 was applied throughout the analysis.

## 4 Results

### 4.1 Common method biases test

To evaluate potential common method variance, Harman's single-factor test was employed through exploratory factor analysis. The results revealed 15 factors with eigenvalues exceeding 1. The first factor explained 29.23% of the total variance, which did not exceed the critical cutoff of 40% (35). Thus, common method bias was not considered a substantial issue in this study.

### 4.2 Preliminary analysis

As displayed in Table 1, medians, interquartile ranges, and bivariate correlations for all study variables are provided. Volunteer motivation demonstrated a significant positive correlation with altruistic behavior (*r* = 0.753, *p* < 0.001) and successful aging (*r* = 0.650, *p* < 0.001). Altruistic behavior was also positively associated with successful aging (*r* = 0.668, *p* < 0.001), while showing a significant negative correlation with loneliness (*r* = −0.460, *p* < 0.001). Furthermore, loneliness was inversely correlated with successful aging (*r* = −0.490, *p* < 0.001).

**Table 1:** Descriptive statistics and correlations of all variables.

**Variables**	**Volunteer motivation**	**Altruistic behavior**	**Loneliness**	**Successful aging**
Volunteer motivation	1			
Altruistic behavior	0.753^**^	1		
Loneliness	−0.397^**^	−0.460^**^	1	
Successful aging	0.650^**^	0.668^**^	−0.490^**^	1
*M*(*P*25, *P*75)	58.00 (53.00, 62.00)	69.00 (62.00, 75.00)	17.00 (15.00, 22.00)	47.00 (41.00, 52.00)

^**^p < 0.01.

### 4.3 Mediating effect of altruistic behavior

To evaluate Hypothesis 2, all variables were standardized, and the mediation analysis was performed using Model 4 from the PROCESS macro (v4.1). As summarized in Table 2, with gender, age, and educational attainment controlled for, volunteer motivation among older adults significantly predicted successful aging (β = 0.630, *p* < 0.001) and altruistic behavior (β = 0.748, *p* < 0.001). Altruistic behavior, in turn, was a significant predictor of successful aging (β = 0.411, *p* < 0.001). The direct effect of volunteer motivation on successful aging remained statistically significant (β= 0.323, *p* < 0.001).

**Table 2:** Results of mediation effect test.

**Variable**	**Model-1-Successful aging**			**Model 2-Altruistic behavior**			**Model 3-Successful aging**		
	β	**se**	***t***	β	**se**	***t***	β	**se**	***t***
Gender	−0.092^*^	0.043	−2.144	0.001	0.037	0.035	−0.092^*^	0.040	−2.308
Age	−0.089^*^	0.043	−2.056	0.001	0.038	−0.006	−0.089^*^	0.040	−2.198
Educational level	−0.036	0.043	−0.838	−0.051	0.038	−1.357	−0.015	0.040	−0.372
Motivations for volunteer services	0.63^***^	0.043	14.569	0.748^***^	0.038	19.720	0.323^***^	0.061	5.311
Altruistic behavior							0.411^***^	0.061	6.767
*R* ^2^	0.44			0.57			0.513		
*F*	60.572^***^			101.958^***^			64.665^***^		

^*^p < 0.05.

^***^p < 0.001.

Further bias-corrected bootstrap analysis based on 5,000 repeated samples indicated a significant mediating effect of altruistic behavior in the relationship between volunteer motivation and successful aging. The direct effect was 0.323 (*p* < 0.001), and the indirect effect mediated by altruistic behavior was 0.307 (*p* < 0.001), accounting for 48.73% and 51.27% of the total effect, respectively. These outcomes provide support for Hypothesis 2.

### 4.4 The moderating effect of loneliness

**Figure 2:**
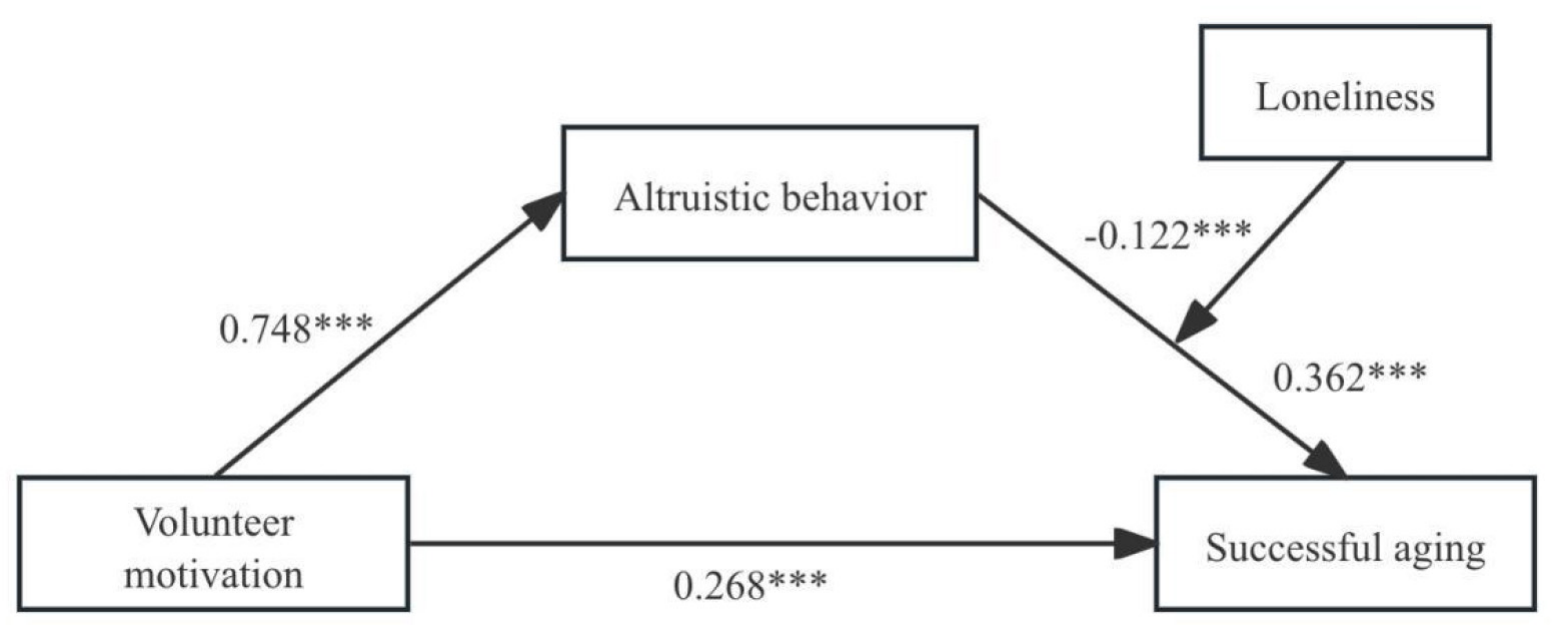
Mediated moderation model. ***p < 0.001.

**Figure 3:**
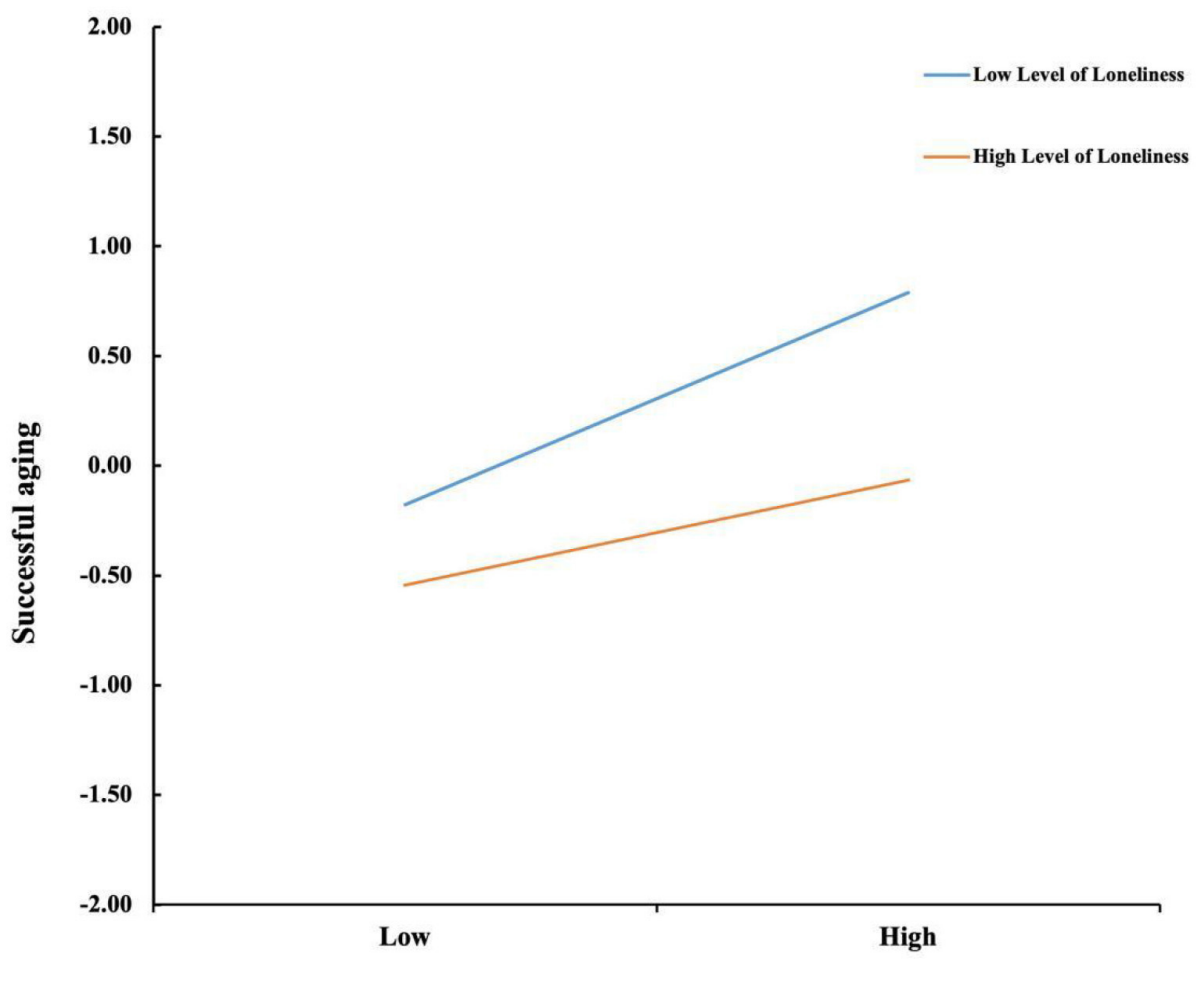
Model of test for simple slopes showing moderating influence of loneliness in the association between altruistic behavior and successful aging.

To examine Hypothesis 3, Model 14 in the PROCESS macro (v4.1) was used to examine whether loneliness moderates the relationship between altruistic behavior and successful aging. As shown in Table 3 and Figure 2, with gender, age, and educational attainment controlled for, the interaction term between altruistic behavior and loneliness significantly negatively predicted successful aging (β = −0.122, *p* < 0.001), indicating a notable moderating effect of loneliness. As presented in Table 4, the simple effects analysis revealed that when loneliness was low (M-SD), altruistic behavior had a stronger effect on successful aging (β = 0.483, *p* < 0.001). When loneliness was high (M+SD), the effect was weaker (β = 0.240, *p* < 0.001), suggesting that the positive association between altruistic behavior and successful aging diminishes as loneliness increases. To visually illustrate this moderating effect, a moderation plot was generated (Figure 3). The pattern was consistent with the simple effects results, confirming the significant negative moderating role of loneliness.

**Table 3:** Results of regulatory effect test.

**Variable**	**Model 4-Altruistic behavior**			**Model 5-Successful aging**		
	β	**se**	***t***	β	**se**	***t***
Gender	0.001	0.037	0.035	−0.097^*^	0.037	−2.580
Age	0.001	0.038	−0.006	−0.081^*^	0.038	−2.126
Educational level	−0.051	0.038	−1.357	−0.063	0.039	−1.617
Motivations for volunteer services	0.748^***^	0.038	19.720	0.268^***^	0.058	4.649
Altruistic behavior				0.362^***^	0.061	5.972
Aloneness				−0.305^***^	0.047	−6.526
Altruistic behavior × Aloneness				−0.122^***^	0.032	−3.844
*R* ^2^	0.570			0.575		
*F*	101.958^***^			59.022^***^		

^*^p < 0.05.

^***^p < 0.001.

**Table 4:** Simple effect test.

**Conditional of loneliness**	**β**	**S.E**.	***t***	***p***	**LLCI**	**ULCI**
M-SD	0.483	0.073	6.576	0.000	0.339	0.628
M	0.362	0.061	5.972	0.000	0.242	0.481
M+SD	0.240	0.063	3.825	0.000	0.116	0.363

To further test whether loneliness moderated the mediating role of altruistic behavior, mediation effect difference tests and the interval test of coefficient products were conducted (36, 37). As shown in Table 5, the bias-corrected bootstrap method (5,000 resamples) indicated that when loneliness was low, the mediating effect of altruistic behavior between volunteer motivation and successful aging was stronger [β = 0.361, 95% CI (0.212, 0.510)]. When loneliness was high, the mediating effect was weaker [β = 0.179, 95% CI (0.049, 0.310)]. Following Hayes' approach for testing moderated mediation (37), the moderated mediation effect coefficient was −0.091, with a 95% CI of [−0.163, −0.022], which did not include zero. Additionally, using Edwards and Lambert's (36) mediation effect difference test, the difference (Diff) was −0.182, 95% CI [−0.325, −0.044], also excluding zero. These results confirm that loneliness significantly moderates the mediating effect of altruistic behavior in the relationship between volunteer motivation and successful aging.

**Table 5:** Results of the test for moderated mediating effect.

**Conditional of loneliness**	**β**	**S.E**.	**95%** ***CI***	
			**Lower**	**Upper**
M-SD (Low)	0.361	0.077	0.212	0.510
M	0.270	0.063	0.150	0.392
M+SD (High)	0.179	0.067	0.049	0.310
Difference (High-Low)	−0.182	0.072	−0.325	−0.044
Index of moderated mediation	−0.091	0.036	−0.163	−0.022

## 5 Discussion

The rapid acceleration of population aging in China presents substantial challenges for social older adult care services and social security systems, including a shrinking labor force and an increasing burden of older adult care. Participation in volunteer activities can help older adults maintain a positive outlook on life, remain socially engaged, and enhance both their physical and mental health. Although a considerable body of empirical research has examined the mechanisms linking older adults' volunteer motivation to successful aging, relatively few studies have explored the mediating role of altruistic behavior in this relationship. Grounded in social exchange theory and productive aging theory, the present study underscores the importance of two key psychosocial factors—altruistic behavior and loneliness—in shaping successful aging. In particular, this research investigates the mediating role of altruistic behavior, as well as the moderating effect of loneliness on the relationship between altruistic behavior and successful aging among older adults.

### 5.1 Volunteer motivation and successful aging

The results of this study revealed a significant positive association between volunteer motivation and successful aging among older adults, confirming Hypothesis 1. This finding aligns with some previous research (20, 38), which indicates that positive volunteer motivation provides older adults with emotional rewards, psychological fulfillment, and opportunities for interaction across different age groups, thereby helping them maintain a positive mindset, achieve personal growth, and ultimately attain successful aging.

However, it is important to note that other studies have reported negative or weakened effects of volunteer motivation on successful aging due to factors such as individual health status and the quality of the volunteer environment. For instance, research by Windsor et al. (39) suggested that advanced age or poor physical health may expose older adults to risks of functional decline during volunteer participation, which could adversely affect their successful aging. Similarly, Wang and colleagues found that the positive impact of volunteer motivation on successful aging was diminished—or even reversed due to frustration—among older adults in remote rural areas, where resource scarcity and poorly structured volunteer programs are common (40).

In light of the above analysis, this study underscores the importance of improving volunteer service mechanisms for the older adults. It is essential not only to provide volunteer opportunities but also to ensure that programs are highly adaptable and capable of delivering benefits to diverse older adult populations, thereby better supporting their successful aging.

### 5.2 The mediating role of altruistic behavior

The findings of this study provide support for Hypothesis 2, indicating that altruistic behavior mediates the relationship between volunteer motivation and successful aging in older adults. This can be explained by the fact that through volunteer services, older adults are able to apply their professional skills and experience to help others and contribute to society (24, 25), thereby gaining social recognition and achieving successful aging. However, some studies have pointed out that when volunteer organizations overemphasize “altruism,” some older adults may withdraw due to perceived role pressure (41, 42). In the pathway to successful aging, these altruistic behaviors also encompass self-interest motivations that similarly influence older adults' volunteer engagement. This is because most older adults, while helping others through volunteering, also seek to attain a sense of personal achievement and social recognition, thereby facilitating successful aging. These research findings form a complementary consensus with the results of this study.

Therefore, it is suggested that when establishing incentive mechanisms for older adult volunteering, efforts should not only emphasize altruistic perspectives but also consider self-interest aspects by highlighting the positive impacts of volunteering on their physical and mental health. Such an approach will better promote the development of a robust volunteer service system for the older adults.

### 5.3 The moderating role of loneliness

The results of this study support Hypothesis 3 and confirm that the sense of loneliness among the older adults moderates the latter part of the pathway through which volunteer motivation influences successful aging, specifically by attenuating the relationship between altruistic behavior and successful aging. Among the older adults with lower levels of loneliness, the influence of altruistic behavior on successful aging is more pronounced. Research has demonstrated that high levels of loneliness in older adults are associated not only with cognitive decline due to physical deterioration, aging anxiety resulting from physiological decline, and ageism, but also with the loss of peer groups, sudden “role loss” following retirement, and the “digital divide” arising from rapid societal development (14, 43). Older adults with higher levels of loneliness often experience more negative emotions, such as anxiety, depression, and helplessness. According to the cognitive model of loneliness, high loneliness drives individuals to become overly focused on their own difficulties, thereby diminishing their ability and willingness to engage in altruistic behavior and ultimately weakening the positive effects that altruistic behavior could have brought (44).

However, some studies have suggested that highly lonely older adults often lack sufficient external support to initiate and sustain altruistic behaviors that could generate strong positive feedback (45). They are more likely to encounter setbacks in the early stages of participation due to internal psychological burdens and external barriers, which inhibits the positive pathway of altruistic behavior. Therefore, loneliness generally plays an inhibitory role in the mechanism linking altruistic behavior and successful aging. Nevertheless, studies on psychological support interventions for the older adults indicate that positive psychological guidance can help highly lonely older adults experience the positive feedback of volunteer services, thereby breaking through their psychological barriers and making volunteer participation an effective way to alleviate loneliness and promote successful aging (46).

Based on the above analysis, it is suggested that society should rapidly improve the volunteer service system for the older adults and create supportive, low-threshold volunteering environments. This would enable older adults suffering from age-related anxiety and other issues to channel their emotions into volunteer activities, helping them overcome their difficulties and achieve successful aging.

## 6 Limitations

This study has several limitations. First, owing to limitations in human, material, and financial resources, a cross-sectional design was utilized, capturing data at a single time point. However, the influence of volunteer motivation on successful aging may involve a dynamic process. Therefore, future studies should employ longitudinal designs to better understand how these relationships evolve over time. Second, the pathway through which volunteer motivation affects successful aging may be influenced by other factors, such as physiological, psychological, and social variables. Future research could further explore how these factors play important roles in elucidating the impact of volunteer motivation on successful aging. Finally, the participant sample was drawn exclusively from several regions within Sichuan Province, China, which may limit the representativeness of the findings. Future investigations would benefit from expanding the geographic coverage and increasing sample sizes to improve representativeness and help develop a more comprehensive understanding of the factors influencing successful aging among older adults in different regions.

## Data Availability

The raw data supporting the conclusions of this article will be made available by the authors, without undue reservation.
